# Distinct clinical outcomes linked to peripheral arthritis and dactylitis in axial spondyloarthritis: findings from a retrospective Irish cohort

**DOI:** 10.1007/s00296-024-05707-0

**Published:** 2024-09-10

**Authors:** Marcus Kenyon, Phil Gallagher, Brona Dinneen, Finbar O’Shea, Ross McManus

**Affiliations:** 1 Department of Rheumatology, St James’ Hospital, Dublin, Ireland; 2 Dept. of Clinical Medicine, Trinity Translational Medicine Institute, James’s St, Dublin, Dublin 8 Ireland

**Keywords:** Axial spondyloarthritis, Ankylosing spondylitis, Dactylitis, Arthritis, Extra-musculoskeletal manifestations

## Abstract

**Introduction:**

Axial spondyloarthritis (AxSpA) is a chronic inflammatory condition primarily affecting the axial skeleton. Peripheral features such as peripheral arthritis (PA) and dactylitis are common in AxSpA disease. This study aimed to investigate the independent impact of these manifestations on patient presentation and disease outcomes within an Irish AxSpA cohort.

**Methods:**

912 Irish AxSpA patients were analyzed in this study. Disease outcomes in patients with and without peripheral arthritis or dactylitis were compared using univariate and multivariate methods. The prevalence of extra-spinal manifestations was further assessed in relation to AxSpA disease duration.

**Results:**

30.2% of patients reported PA, while 6.6% had dactylitis. PA and dactylitis were strongly linked, with 70% of patients presenting with dactylitis also showing features of PA. Psoriasis was more common in both patients with PA (OR 2.2, *P* < 0.001) and dactylitis (OR 3.38, *P* < 0.001). Dactylitis, but not PA was strongly linked to uveitis (OR 2.91, *P* < 0.001) and inflammatory bowel disease (OR 3.15, *P* < 0.001), while PA was associated with worse patient functioning and reduced quality of life. PA, but not dactylitis was linked with increased AxSpA disease duration.

**Discussion:**

Despite high concurrence of PA and dactylitis in AxSpA patients, each manifestation is independently associated with worse outcomes. While some of these overlapped, several outcomes are specific to either PA or dactylitis. Due to its strong association with uveitis and inflammatory bowel disease, an early presentation of dactylitis may represent a unique subset of patients and serve as a valuable predictive marker for the later onset of these conditions.

## Key points

Peripheral arthritis and dactylitis are closely correlated but independent manifestations in AxSpA, each uniquely contributing to increased disease severity.

Dactylitis is uniquely associated with increased risk of uveitis and inflammatory bowel disease, while peripheral arthritis is uniquely linked with depression.

An early presentation of dactylitis in AxSpA patients may be predictive of later uveitis episodes.

## Introduction

Axial Spondylarthritis (AxSpA) is a chronic rheumatic inflammatory disease characterised by inflammation of the lumbar spine and sacroiliac joints. In addition to spinal involvement, AxSpA may present with peripheral arthritic symptoms, including peripheral arthritis (PA), enthesitis and dactylitis, together with a range of extra-musculoskeletal manifestations (EMMs). The presence of peripheral involvement is a significant clinical finding which may impact on both the severity of AxSpA disease and the decision making process for selecting appropriate therapies [1].

Peripheral arthritis and dactylitis are peripheral features of AxSpA which are closely related to each other. PA is a common manifestation reported in AxSpA patients, and although wide variations in prevalence, ranging 18–58% are reported by individual studies, its overall prevalence in radiographic of AxSpA is approximately 23% [2]. Dactylitis is a form of peripheral enthesitis which is characterized by complete or proximal diffuse digital swelling (sausage-shaped digits). It is observed in a much smaller proportion (around 6%) of AxSpA patients than peripheral arthritis [2, 3]. However, a strong overlap exists between these manifestations, with the prevalence of dactylitis being up to 9 times higher in AxSpA patients with PA than in those without [4]. Despite this strong correlation, there remains proportions of patients who present with either PA or dactylitis in isolation. The potential for patients initially presenting with one manifestation to eventually develop the other remains unclear, as no follow-up studies have specifically examined the concurrence of dactylitis and peripheral arthritis. However, the prevalence of PA in AxSpA has been shown to increase over a 20 year follow-up period [5], suggesting that at least a proportion of patients with earlier presentation with dactylitis will eventually develop concomitant arthritis. Conversely, a 5 year follow-up from the DESIR cohort showed that the prevalence of dactylitis remained steady over this period, implying that dactylitis is less likely to occur later in AxSpA disease [2, 4].

Of these two manifestations, PA remains the best described in the context of AxSpA. Studies of AxSpA and SpA as a whole indicate that PA is more often observed in older patients and has a female predilection [4, 6]. It is also more frequent in HLA-B27 negative disease and in never smokers [4, 6]. PA is robustly linked with worse patient outcomes in AxSpA, including increased risk of psoriasis, raised C-reactive protein and worse outcomes as measured by patient reported outcomes (PROs) for disease activity and functioning [4, 7]. However, there is some evidence to support a reduced risk of uveitis in AxSpA patients with PA [4].

Limited research has been conducted to explore the clinical relevance of dactylitis in terms of AxSpA disease presentation and severity. A notable study identified a connection between dactylitis and other peripheral manifestations, including enthesitis and psoriasis, as well as increased inflammation [8]. However it is not clear whether these symptoms are caused by the frequently co-occurring PA or are independently associated with (and potentially caused by) dactylitis specific disease processes.

In the present study we aimed to evaluate the significance of PA and dactylitis in terms of AxSpA disease severity and the occurrence of additional peripheral features within a large Irish AxSpA cohort. Furthermore, we aimed to disentangle the disease associations of dactylitis and PA, which are often concurrent manifestations, independently contribute to patient outcomes.

## Methods

### Study design

Data for this study were obtained from the Ankylosing Spondylitis Registry of Ireland (ASRI), a multi-center, cross-sectional cohort study focusing on Axial Spondylarthritis (AxSpA). The registry was established in 2013 with the primary objective to identify predictive factors of disease outcome. ASRI collects comprehensive demographic and phenotypic information from enrolled individuals.

Patients eligible for recruitment are over 18 years old and have received a diagnosis of AxSpA based on the modified New York (mNY) criteria or the Assessment of SpondyloArthritis International Society (ASAS) criteria for AxSpA from a practising rheumatologist. Additionally, participants must have attended secondary or tertiary care within the preceding 3 years. Patients with any impairment which may prevent informed consent are excluded. Patients diagnosed with AxSpA who received diagnosis with other spondyloarthropathy were excluded. Each participating centre has a designated individual responsible for local oversight of the study. Overall responsibility for oversight is managed by the principal investigator (author FOS).

### Data collection

Written informed consent was obtained from all participants. Data from the study participants were collected using a structured interview format conducted by trained professionals. Information was obtained through clinical assessments performed during registration or self-reporting if clinical records were unavailable.

The collected data encompassed various aspects, including demographic information such as age, sex, ethnicity, and family history of AxSpA. Clinical characteristics recorded included self-reported age of disease onset, age of diagnosis, presence of extra-axial manifestations, comorbidities, HLA-B27 status, conformity with the modified New York (mNY) criteria, and compliance with the Assessment of SpondyloArthritis International Society (ASAS) criteria for axial spondyloarthritis. Additionally, information regarding current therapies was documented.

Physical examination included modified Schober test, tragus to wall distance, lumbar side flexion, intermalleolar distance, cervical rotation and chest expansion. All of these examinations were performed in accordance with standardised technique [9].

Validated outcome measures used to assess patients included:

Bath Ankylosing Spondylitis Disease Activity Index (BASDAI): measured in the range 0–10. Higher scores indicate more severe disease [10].

Bath Ankylosing Spondylitis Functional Index (BASFI): measured in the range 0–10. Higher scores indicate worse functioning [11].

Bath Ankylosing Spondylitis Metrology Index (BASMI): measured in the range 0–10. Higher scores reflect worse spinal mobility [9].

Ankylosing Spondylitis Quality of Life (ASQoL): measured in the range 0–18. Higher scores indicate worse quality of life [12].

Health Assessment Questionnaire modified for the Spondyloarthropathies (HAQ-S): assessed on a scale of 1–3. Higher scores indicate greater disability [13].

Disease activity was assessed using the BASDAI. Disease severity and functionality were evaluated using the BASMI and BASFI, respectively. Quality of life and disability were measured using the ASQoL and the HAQ-s questionnaires, respectively.

HLA-B27 types were obtained from genotyping data, where available. Among the 912 patients included in the study, 641 had available genotyping data for HLA-B27 status. For the remaining patients, HLA types were derived from the ASRI database records, obtained from medical records, or self-reported, if available.

Not all patients had all measurements recorded; patients with missing data were excluded from respective analyses.

### Statistical analysis

The collected data were formatted for analysis using Microsoft Excel. Descriptive statistics are presented as median with interquartile range or frequencies with percentage, as appropriate.

Statistical analyses were performed using Minitab V21.1 software. For comparison of groups, the Mann Whitney U test or Kruskal-Wallis test was employed for continuous data, depending on the number of groups being compared. Proportions were analyzed using Fisher’s exact test.

Regression analyses, including logistic regression, binary logistic regression, and multivariate regression, were conducted using Minitab V21.1. Covariates included in multivariate models included age, gender, HLA-B27 status, Axial Spondylarthritis (AxSpA) disease duration, smoking status (categorized as current, past, or never), and BMI.

Results from all analyses were considered statistically significant where *P* < 0.05. All graphical representations of the data were created using Minitab V21.1 software.

## Results

At the time of analysis, data from 912 patients were included in the study. Table 1 provides a summary of the characteristics of these patients.

**Table 1:** General characteristics of the ASRI cohort

	Included *n*	Median (IQR) or *n*(%)	Range
**Demographics and disease characterisation**			
Gender (male %)	912	75.1	--
Age, median (IQR)	912	44 (36–55)	18–85
Age at AxSpA symptom onset, median (IQR)	906	25 (19–33)	7–78
Age at diagnosis, median (IQR)	906	34 (26–42)	9–80
Delay to diagnosis in years, median (IQR)	906	6 (2–11)	0–52
Disease duration in years, median (IQR)	912	16 (9–27)	0–57
ASAS criteria for AxSpA, *n* (%)	912	912 (100)	--
mNY criteria for AS, *n* (%)	912	694 (76.1)	--
HLA-B27 positive, *n* (%)	863	733 (84.9)	--
**Clinical measures of disease activity and severity**			
BASDAI score, median (IQR)	912	3.7 (1.9–5.8)	0.0–10.0
BASMI score, median (IQR)	912	3.6 (2.4–5.6)	0.4–9.0
BASFI score, median (IQR)	912	3.2 (1.3–5.6)	0.0–10.0
AsQoL score, median (IQR)	912	5.0 (1.0–11.0)	0.0–18.0
HAQ-s score, median (IQR)	912	0.4 (0.0–0.8)	0.0–3.0
**Peripheral manifestations and EMMs**			
Uveitis, *n* (%)	895	308 (34.4)	--
Peripheral arthritis, *n* (%)	894	276 (30.9)	--
Psoriasis, *n* (%)	895	150 (16.8)	--
IBD^1^, *n* (%)	897	96 (10.7)	--
Dactylitis, *n* (%)	897	60 (6.7)	--
Depression, *n* (%)	905	94 (10.4)	--
**Current therapies**			
Anti-TNF, *n* (%)	912	531 (58.2)	--
IL-17 pathway inhibitor, *n* (%)	912	14 (1.5)	--
Methotrexate, *n* (%)	903	48 (5.3)	--
Sulfasalazine, *n* (%)	903	33 (3.7)	--
NSAID, *n* (%)	903	461 (51.1)	--
Multiple therapies, *n* (%)	912	275 (30.2)	--
No current therapy, *n* (%)	912	127 (13.9)	--

^1^ Defined as ulcerative colitis or Crohn’s disease

Within the ASRI cohort, 685 patients (75.1%) were male. The median age of the participants was 44 years, and the median age at the onset of the first Axial Spondylarthritis (AxSpA) symptom was 25 years. The average disease duration among the participants was 16 years.

A majority of the patients, 694 (76.1%), met the modified New York (mNY) criteria for radiographic AxSpA (rAxSpA), while 733 patients (84.9%) were found to be HLA-B27 positive. Frequently reported EMMs included uveitis (34.4%), psoriasis (16.8%), inflammatory bowel disease (10.7%), and depression (10.4%).

In terms of treatment, the most frequently administered therapies were tumor necrosis factor (TNF) inhibitors (58.2%) and non-steroidal anti-inflammatory drugs (NSAIDs), which were used by 51.1% of participants. 30.2% of participants were receiving multiple treatment options, while 13.9% were not receiving any treatment.

### Features associated with PA and dactylitis

Out of the 912 patients, 276 participants (30.9%) reported peripheral arthritis (PA), while 60 patients (6.6%) had dactylitis. Table 2 provides a summary of the characteristics associated with these peripheral manifestations.

**Table 2:** Characteristics of AxSpA patients with peripheral arthritis and/or dactylitis

	Peripheral arthritis					Dactylitis				
	Yes (*n* = 276)	No (*n* = 618)	OR	*P*-value^b^	Multiple regression^c^	Yes (*n* = 60)	No (*n* = 837)	OR	*P*-value^b^	Multiple regression^c^
**Demographics and disease characteristics**										
Male sex, *n* (%)	193 (69.9)	478 (77.3)	0.68	**0.019**	--	33 (55.0)	638 (76.2)	0.38	**0.003**	--
Age in years, median (IQR)	48 (38–57)	44 (35–53)	--	**< 0.001**	--	46 (37–55)	44 (36–55)	--	0.618	--
Age at AxSpA symptom onset, median (IQR)	25 (19–33)	24 (19–33)	--	0.956	--	24 (19–31)	25 (19–33)	--	0.6	--
Age at diagnosis, median (IQR)	35 (25–43)	33 (27–42)	--	0.884	--	36 (26–43)	34 (27–42)	--	0.355	--
Delay to diagnosis in years, median (IQR)	5 (2–11)	6 (2–12)	--	0.342	--	7 (3–18)	6 (2–11)	--	0.331	--
Disease duration in years, median (IQR)	19 (10–31)	15 (9–26)	--	**0.001**	--	18 (11–28)	16 (9–27)	--	0.407	--
mNY criteria for AS, *n* (%)	201 (72.8)	477 (77.2)	0.79	0.176	--	35 (58.3)	645 (77.1)	0.41	**0.003**	--
HLA-B27 positive, *n* (%)	215 (83.0)	503 (85.7)	0.81	0.349	--	46 (80.7)	677 (85.2)	0.78	0.343	--
**Clinical measures of disease severity**										
BASDAI score, median (IQR)	4.4 (2.2–6.3)	3.3 (1.8–5.6)	--	**< 0.001**	**0.007**	5.6 (3.7–7.1)	3.5 (1.8–5.7)	--	**< 0.001**	**0.002**
BASMI score, median (IQR)	3.8 (2.4–5.6)	3.6 (2.2–5.6)	--	0.153	--	3.3 (2.6–5.3)	3.6 (2.2–5.6)	--	0.942	--
BASFI score, median (IQR)	3.7 (1.5–6.4)	2.9 (1.2–5.2)	--	**0.001**	**0.005**	3.9 (1.7–6.4)	3.1 (1.3–5.5)	--	0.111	--
AsQoL score, median (IQR)	7.0 (2.0–13.0)	4.0 (1.0–10.0)	--	**< 0.001**	**0.002**	8.5 (2.8–13.0)	5.0 (1.0–11.0)	--	**0.011**	0.062
HAQ-s score, median (IQR)	0.5 (0.1–1.0)	0.4 (0.0–0.8)	--	**< 0.001**	**< 0.001**	0.6 (0.2–1.0)	0.4 (0.0–0.8)	--	**0.023**	0.222
**Peripheral manifestations and EMMs**										
Uveitis, *n* (%)	113 (42.3)	190 (30.8)	1.56	**0.01**	0.068	35 (59.3)	272 (32.6)	2.91	**< 0.001**	**< 0.001**
Psoriasis, *n* (%)	68 (25.2)	80 (13.0)	2.20	**< 0.001**	**0.001**	21 (35.6)	126 (15.1)	3.38	**< 0.001**	**0.006**
IBD^a^, *n* (%)	38 (14.0)	57 (9.3)	1.57	**0.045**	0.636	15 (25.0)	80 (9.6)	3.15	**0.001**	**0.007**
Peripheral arthritis, *n* (%)	--	--	--	--	--	42 (70.0)	226 (27.4)	6.31	**< 0.001**	--
Dactylitis, *n* (%)	42 (15.7)	18 (2.9)	5.98	**<<0.001**	--	--	--	--	--	--
Depression, *n* (%)	39 (14.2)	52 (8.5)	1.79	**0.012**	**0.026**	6 (10.2)	85 (10.2)	0.98	--	--
**Current therapies**										
Anti-TNF, *n* (%)	171 (62.0)	350 (56.6)	1.25	0.143	--	34 (56.7)	486 (58.1)	0.94	0.893	--
Methotrexate, *n* (%)	32 (11.7)	16 (2.6)	4.93	**< 0.001**	**< 0.001**	10 (16.9)	37 (4.5)	4.32	**0.001**	**0.001**
Sulfasalazine, *n* (%)	16 (5.9)	16 (2.6)	2.31	**0.03**	**0.002**	3 (5.1)	29 (3.5)	1.47	--	--
NSAID, *n* (%)	152 (55.7)	297 (48.5)	1.32	0.058	--	33 (55.9)	421 (50.8)	1.20	0.501	--
Multiple therapies, *n* (%)	110 (39.9)	157 (25.4)	1.95	**< 0.001**	**< 0.001**	21 (35.0)	247 (29.5)	1.29	0.383	--
No current therapy, *n* (%)	30 (10.9)	94 (15.2)	0.68	0.094	--	5 (8.3)	119 (14.2)	0.55	--	--

^a^Defined as ulcerative colitis or Crohn’s disease

^b^Mann Whitney U or Fisher’s exact

^c^Includes gender, HLA-B27, AxSpA disease duration, smoking status, BMI and dactylitis or peripheral arthritis as covariates

There was a strong association between peripheral arthritis and dactylitis within the cohort. Among the 60 patients with dactylitis, 42 (70%) also presented with peripheral arthritis, indicating a significant overlap between the two conditions (OR 6.31).

In univariate analysis, both peripheral arthritis (OR 0.68, *P* = 0.019) and dactylitis (OR 0.38, *P* = 0.003) were inversely associated with male sex. Peripheral arthritis was associated with older patient age (48 vs. 44 years, *P* < 0.001) and longer AxSpA duration (19 vs. 15 years, *P* < 0.001). However, no relationship was observed between dactylitis and either age or AxSpA disease duration.

Patients with PA or dactylitis had worse BASDAI scores compared to those without these peripheral features (PA: 4.4 vs. 3.6, *P* < 0.001; dactylitis: 5.6 vs. 3.5, *P* < 0.001). These associations remained robust in multivariate regression analysis. Peripheral arthritis, but not dactylitis, showed residual association with worse scores in BASFI (3.7 vs. 2.9, *P* = 0.005), AsQoL (7 vs. 4, *P* = 0.002), and HAQ-s (0.5 vs. 0.4, *P* < 0.001), after accounting for covariates in multivariate analysis.

Among the EMMs, psoriasis was more frequent in patients with both PA (OR 2.2, *P* < 0.001) and dactylitis (OR 3.38, *P* < 0.001). However, while dactylitis showed a strong association with uveitis (OR 2.91, *P* < 0.001) and inflammatory bowel disease (IBD) (OR 3.15, *P* < 0.001), PA did not show a significant association with either of these manifestations after accounting for covariates. PA was associated with accompanying depression (OR 1.79, *P* = 0.012), which remained significant after accounting for covariates.

Therapeutic options chosen by clinicians differed based on the presence of PA and dactylitis in patients. Methotrexate was more commonly administered to patients with PA (OR 4.93, *P* < 0.001) and/or dactylitis (OR 4.32, *P* = 0.001). Peripheral arthritis was also associated with the administration of sulfasalazine (OR 2.31, *P* = 0.03) and multiple drug treatments (OR 1.95, *P* < 0.001). While sulfasalazine and multiple therapies were also, to a lesser degree, more commonly administered to patients with dactylitis (OR 1.47 and 1.29, respectively), these findings were not statistically significant.

### Peripheral arthritis but not dactylitis is associated with AxSpA disease duration

**Fig. 1:**
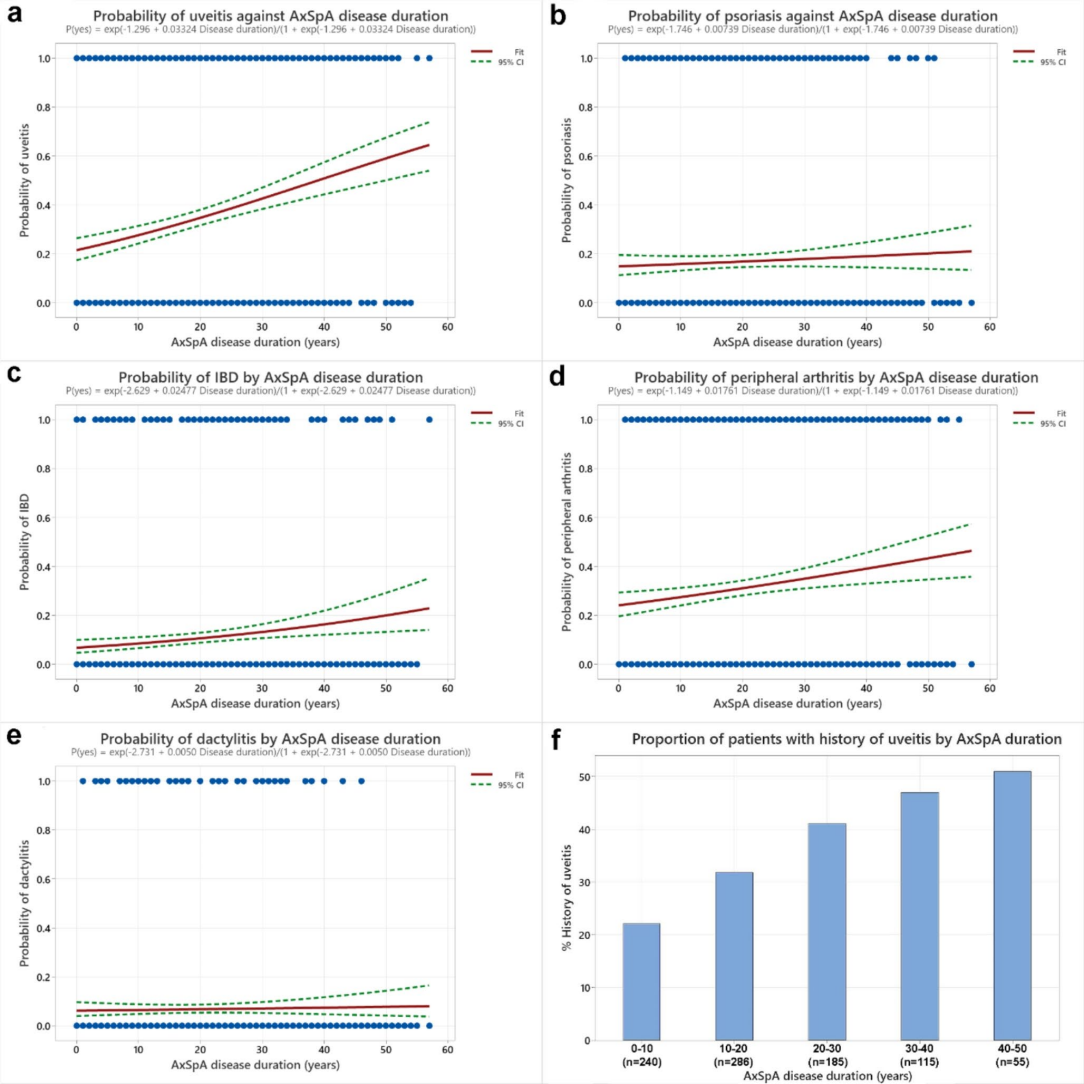
(**a-e**) Binary logistic regression of peripheral features against AxSpA disease duration. Trend indicated in red; dashed lines represent 95% confidence interval. (**f**) Bar chart representation of the proportion of patients with a history of uveitis against AxSpA disease duration

Due to the lack of specific dates when peripheral manifestations developed in patients, we employed logistic regression to model the presence or absence of these manifestations against AxSpA disease duration. The results of this analysis are presented in Fig. 1 (a-e).

All of uveitis, peripheral arthritis, and inflammatory bowel disease (IBD) showed a positive association with AxSpA disease duration. Particularly, uveitis exhibited a notable trend, with its prevalence reaching 50% after 40–50 years post-AxSpA onset (Fig. 1f). In contrast, both psoriasis and dactylitis demonstrated no significant association with AxSpA disease duration, suggesting that these features tend to either precede or develop shortly after AxSpA onset.

## Discussion

In this study we have demonstrated that PA and dactylitis are very closely related manifestations with a strong tendency to coincide. As much as 70% of patients who present with dactylitis may have concomitant PA. Despite this close relationship, we have shown that each of these discrete manifestations is independently associated with worse disease outcomes in AxSpA patients.

Consistent with previous findings from the ASAS COSMOSPA and a 5-year follow-up of the DESIR cohort, our study found a female predominance in peripheral arthritis [4, 6]. This trend was also observed in the presentation of dactylitis within our cohort, contrasting with the ESPeranza cohort’s results [8]. This discrepancy could be attributed to the ESPeranza cohort’s inclusion of all SpA subtypes.

Moreover, both manifestations were correlated with increased disease activity. Specifically, PA was associated with worse functioning, as measured by BASFI and HAQ-s, and worse quality of life in patients. Depression is an additional risk identified in patients presenting with PA, which is, perhaps, not surprising given its association with worse functioning in patients. It appears likely that dactylitis is also linked to worse functioning in patients, although this was not statistically significant after the presence of PA was accounted for. Both uveitis and IBD were present with approximately three times higher odds in patients with dactylitis. This correlation withstood multivariate regression analysis which accounted for concurrent peripheral arthritis in patients. These findings differ from those of the ESPeranza cohort, which reported no significant association [8]. This discrepancy could be due to their focus on early SpA disease, as evidenced by the low incidence (4.6%) of uveitis—a characteristic typically associated with longer established SpA—reported in their study. A positive correlation between dactylitis and uveitis was also recorded in a recently published report from the Chinese Spondyloarthritis Registry [14]. No residual association of either uveitis or IBD with PA was observed in our models after concurrent dactylitis was accounted for, this being consistent with previous studies [4, 6]. This indicates a unique association between dactylitis and both uveitis and IBD which can not be accounted for by concomitant PA.

Contrary to previously published findings which showed a marked increase in HLA-B27 negative disease amongst patients presenting with peripheral arthritis [4] and dactylitis [15], we did not find a statistically significant difference in HLA-B27 positivity in patients with either PA or dactylitis, although we noted that HLA-B27 carriage rates were lower amongst patents with these manifestations. Dactylitis, but not peripheral arthritis was associated with non-radiographic AxSpA. Our findings are consistent with previously published results by Londono [16], the German Spondyloarthitis inception cohort [17] and SPACE cohort [18]. Notably, no association between dactylitis and non-radiographic AxSpA was reported in either the ASAS-COSMOSPA [19] or DESIR [20] cohorts.

Both PA and dactylitis are important clinical findings which have impacts on the choices of therapy chosen by physicians, with methotrexate being a key drug of choice in the treatment of both of these manifestations. This increased the likelihood of such patients receiving multiple therapeutic treatments. Previously published findings from the DESIR cohort reported increased use of TNF inhibitors in patients with peripheral arthritis [4]. In our study, we observed a slightly higher proportion of patients with peripheral arthritis receiving anti-TNF therapy, although this was not statistically significant. Additionally, we found no evidence of increased anti-TNF therapy use in patients with concomitant dactylitis.

Although we lacked the dates of onset of peripheral features within our cohort, we demonstrated that the prevalence of uveitis, PA and IBD all increase with AxSpA disease duration using regression models. These findings are in line with the previous reports [5, 21, 22].

Contrary to the findings of Zeboulon et al. (2008), we did not observe a plateau of uveitis prevalence at 20 years post-AxSpA onset within the ASRI cohort. Instead, the prevalence of uveitis continued to rise at a relatively steady rate with disease duration with the primary limitation being survivorship. In contrast, dactylitis showed no relationship with AxSpA disease duration, instead maintaining a stable prevalence of approximately 6%. This suggests that the onset of dactylitis is more likely to occur either early following, or possibly preceding AxSpA onset. This perspective aligns with previous findings from the DESIR cohort, which demonstrated no increase in the prevalence of dactylitis over a five-year period [2, 4].

Given its strong association with uveitis, PA and IBD, a presentation of dactylitis may offer predictive value in the subsequent onset of these EMMs in AxSpA patients. Interestingly, dactylitis was, at the same time, inversely correlated with radiographic AxSpA and male sex, both of which are classically positive predictors of uveitis [2]. This suggests that AxSpA patients presenting with dactylitis may represent a unique sub-cohort with increased peripheral symptoms and reduced axial involvement.

### Study limitations

This study was limited by cross sectional design. Models describing the likelihood of various manifestations against AxSpA duration lacked dates of onset of these additional manifestations. This limits our ability to assess cause and effect within the cohort.

Due to the limited number of dactylitis patients within our cohort and strong correlation with PA, we were unable to accurately model the relationship between dactylitis and either uveitis or IBD with respect to AxSpA disease duration. Therefore, it would be interesting to observe how the course of AxSpA disease progression differs between patients with and without dactylitis in the context of a longitudinal study.

## Declarations

### Congress abstract publication

This work has not been showcased in any congress at the time of publication.
